# Review Department

**Published:** 1889-04

**Authors:** 


### REVIEW DEPARTMENT.

THE TROPICAL DISEASES OF THE HORSE. By R. W. BURKE, M.R.C.V.S., A.V.D., author of a "Text-Book on Veterinary Surgical Pathology." Second edition. Imperial Press, Jubbulpore, 1888.

This is a welcome echo of the progress which veterinary science has been making of late in the distant East. The author is already well known by his "Text-Book on Veterinary Surgical Pathology," and scarcely needs a word of commendation in America.

He has devoted his attention in these pages to a consideration of equine disease as it is influenced by the climatic and other conditions of India, and though this fact would seem to rob the book of a share of its general interest to the veterinarian the world over, yet such indeed is far from being the case. The author has viewed his subject from a strictly scientific standpoint, and has thus been able to refer peculiar and exceptional pathological conditions to the operation of general principles. The various forms of anthrax among the horses of India exhibit the same general outlines as those in Germany and France, only that in the case of the furious and paralytic orders the fatality is greater and more rapid in the East. The author's observations on the course and treatment of this disease are brief and lucid, and cover the same ground as more elaborate treatises. He is strongly in favor of protective inoculation, and deems it the duty of every government to take proper and effective measures to this end. The chapter on "Surra," or pernicious anæmia, is highly interesting, and comes fully up to the plane reached by the most recent scientific enquiries. What the author says on "Tropical Pitzriasis" is more distinctly local, and exhibits a phase of morbid processes peculiar to the East. The enervating heat of the country is the main cause of this form of skin disease. Dr. Burke's treatise is in a clear and readable style, and will everywhere meet a favorable reception from veterinarians. C. M. O'L.

---

VETERINARY LECTURES (THIERARZTLICHE VORTRAGE) 1888. Vol. I. Fasciculi 1–6. Published by the Editor, Dr. Schneidemühl, Halle *a.d.* Saale, Germany.

We have before us the inaugural number of a new German veterinary periodical, issued by Dr. Schneidemühl, of Halle, in Prussia, which, if it fulfills the promise of its first pages, is destined to occupy a high plane in veterinary journalism. Somewhat after the fashion of Volkmann's celebrated clinical lectures, it consists of a series of lectures on veterinary subjects. The first is on bacillary erysipelas and the hog epizootic, by Prof. E. Hess of Bern. He states that the term hog erysipelas is an *omnium gatherum*, and that at least two distinct diseases, *i. e.*, bacillary erysipelas of the pig and the ordinary swine plague must be separated from each other. He reports that as regards the former disease Pasteur's much vaunted prophylactic vaccination did not yield him very favorable results, of forty-six hogs protectively (sic !) vaccinated in 1885. Eleven sickened before the sixth day of acute erysipelas, and three of them died, while others remained behind in development. The second lecture by Prof. R. Edelmann of Dresden, deals with the recent progress in studying the digestive processes in domestic animals. This paper is worthy of being translated, presenting an able summary of facts with which the majority of American and English veterinarians have had but little opportunity to familiarize themselves. Müller's lecture, the third is an able exposition of the application of antiseptic surgery to veterinary practice, while the fourth on emphysema contagiasmn, a variety of anthrax not yet reported in this country has a purely theoretical interest. Prof. Hoffmann's paper on general and local anæsthesia for veterinary operative purposes closes the series. It strikes us that veterinary practice has not yet reached that pinnacle of æstheticism, justifying long disquisitions on hypnotics such as ether, and other drugs whose expense is in inverse relation to their usefulness. E. C. S.

